# An End-to-End Image-Based Automatic Food Energy Estimation Technique Based on Learned Energy Distribution Images: Protocol and Methodology

**DOI:** 10.3390/nu11040877

**Published:** 2019-04-18

**Authors:** Shaobo Fang, Zeman Shao, Deborah A. Kerr, Carol J. Boushey, Fengqing Zhu

**Affiliations:** 1 School of Electrical and Computer Engineering, Purdue University, West Lafayette, IN 47907, USA; fang29@purdue.edu (S.F.); shao112@purdue.edu (Z.S.); 2 School of Public Health, Curtin University, Perth, WA 6845, Australia; d.kerr@curtin.edu.au; 3 Curtin Institute of Computation, Curtin University, Perth, WA 6845, Australia; 4 Cancer Epidemiology Program, University of Hawaii Cancer Center, Honolulu, HI 96813, USA; cjboushey@cc.hawaii.edu; 5 Department of Nutrition, Purdue University, West Lafayette, IN 47907, USA

**Keywords:** dietary assessment, food energy estimation, generative models, generative adversarial networks, image-to-energy mapping, neural networks, regressions

## Abstract

Obtaining accurate food portion estimation automatically is challenging since the processes of food preparation and consumption impose large variations on food shapes and appearances. The aim of this paper was to estimate the food energy numeric value from eating occasion images captured using the mobile food record. To model the characteristics of food energy distribution in an eating scene, a new concept of “food energy distribution” was introduced. The mapping of a food image to its energy distribution was learned using Generative Adversarial Network (GAN) architecture. Food energy was estimated from the image based on the energy distribution image predicted by GAN. The proposed method was validated on a set of food images collected from a 7-day dietary study among 45 community-dwelling men and women between 21–65 years. The ground truth food energy was obtained from pre-weighed foods provided to the participants. The predicted food energy values using our end-to-end energy estimation system was compared to the ground truth food energy values. The average error in the estimated energy was 209 kcal per eating occasion. These results show promise for improving accuracy of image-based dietary assessment.

## 1. Introduction

Leading causes of death in the United States, including cancer, diabetes, and heart disease, can be linked to diet [1,2]. Measuring accurate dietary intake is considered to be an open research problem, and developing accurate methods for dietary assessment and evaluation continues to be a challenge. Underreporting is well documented amongst dietary assessment methods. Compared to traditional dietary assessment methods that often involve detailed handwritten reports, technology-assisted dietary assessment approaches reduce the burden of keeping such a detailed report and are preferred over traditional written dietary record for monitoring everyday activity [3].

In recent years, mobile telephones have emerged and provide unique mechanisms to monitor personal health and to collect dietary information [4]. Image-based approaches integrating application technology for mobile devices have been developed which aim at capturing all eating occasions by images as the primary record of dietary intake [3]. To date, these image-based approaches have primarily relied on trained analysts to estimate energy intake from the food images. Validation studies of the trained analyst have shown limited accuracy within and between the trained analysts [5,6]. Although automated methods are not sufficiently advanced to entirely replace the trained analyst, these methods hold promise to ultimately improve accuracy and reduce participant and researcher burden. Several mobile dietary assessment systems have been developed, such as the Technology Assisted Dietary Assessment (TADA^TM^) system [7,8], FoodLog [9], FoodCam [10], DietCam [11], and Im2Calories [12], to address some of the challenges of automatically-determined food types and energy consumed based on image processing and analysis methods. However, developing automatic dietary assessment techniques remains an open research problem.

Estimating food energy from a single-view food image is an ill-posed problem, as most 3D information has been lost when the eating scene is projected from 3D world coordinates onto 2D image coordinates. Several methods have been proposed to estimate food portions from a single-view image. In Chen et al. [13], 3D models were manually fitted onto a 2D food image in order to estimate the food portion sizes. However, manual fitting does not scale with larger data sets. Another method used was participants placing their thumbs in their images as a size reference to estimate the food area and then the portion size of the food [14]. The inconsistency in the sizes of thumbs is an obvious issue. The model proposed by Zhang et al. [15] counts the pixels of each food segmentation in the image to estimate food portion. No 3D information is incorporated into the model. In the approach used by Aizawa et al. [16], the food image is divided into sub-regions and then food portions are estimated based on predetermined serving size classifications. Food portion estimation, in this case, is a task of selecting from limited discrete portion size choices.

We previously developed a 3D geometric-model based method for food portion estimation [17]. Our technique did not require manual tuning of model parameters, and we were able to obtain accurate food portion estimates [17]. Later, we showed that accurate food portions could be estimated using geometric models for food objects with well-defined 3D shapes [18]. To further improve the accuracy of food portion estimation, we incorporated the contextual dietary information of food portion co-occurrence patterns [19]. However, geometric-model-based techniques estimate food volumes rather than food energy. With food volumes estimated, food density is still required to compute the food weights which can then be mapped to food energy using a food composition resource, such as, the United States Department of Agriculture (USDA) Food and Nutrient Database for Dietary Studies (FNDDS) [20]. In addition, geometric-model-based techniques require food labels and food segmentation masks (i.e., location of foods in the image). Errors from automatic food classification and image segmentation can propagate into the final portion estimation. Therefore, new approaches that can directly link food images to food energy in the image would be desirable.

Recently, deep learning [21] techniques, especially techniques based on Convolutional Neural Networks (CNN) [22] have shown substantial success in many computer vision techniques, such as object detection [23,24,25], object segmentation [26], and image to image transfer [27,28,29]. Meyers et al. [12] proposed a food portion estimation method based on the predicted depth maps [30] of the eating scene. We have shown there is a tendency of over-estimation using depth image-based techniques, and an accurate estimation is not always guaranteed, even when depth information is available [18]. Ege et al. [31] used a multi-task CNN [32] architecture for identification of food, ingredients, and cooking directions. Food energy estimation is treated as a regression task [31], and only one unit in the last fully-connected layer in the VGG-16 architecture [23] is used for energy estimation. Further analysis of where the error may come from for energy estimation becomes difficult. Techniques based on CNN rely highly on well-constructed training data sets with sufficient samples and properly designed neural network architecture. In this paper, we focused on automatic dietary assessment of food energy estimation. We used single-view food images captured by users before and after eating their meals.

We proposed the concept of an “energy distribution image”, which was one approach to establish the relationships between the food image and how food energy was distributed in the food image [33]. Each pixel in the energy distribution image represented the relative food energy weights at the corresponding pixel location. The use of an “energy distribution image” enabled us to first visualize how food energy estimation was spatially distributed across the eating scene.

Generative models learn from real data distribution and can generate samples that are similar to those in the real data distribution by taking random noises (for example, generate fake faces that look realistic [34]). In addition, generative models can also take prior information when generating new samples [27]. Therefore, they are suitable for tasks of image-to-image translation. We used generative models to predict energy distribution image based on eating occasion image, as generative models are a natural fit for solving image-to-image translations by its proven capability of learning the correspondences from one data distribution to another [27]. The aim of this paper was to develop a novel dietary assessment method to estimate the food energy numeric value from eating occasion images.

## 2. Methods

To estimate food portions (in energy), the energy distribution image is a new approach to visualize where foods are in the image and how much relative energy is presented at different food regions. We used Generative Adversarial Network (GAN) architecture to train the generative model that predicts the food energy distribution images based on eating occasion images. We built a food image data set with paired images for the training of the GAN [33]. To complete the end-to-end task of estimating food energy value based on a single-view eating occasion image, we used a CNN based regression model to estimate the numeric food energy value using the learned energy distribution images.

### 2.1. Image-to-Energy Data Set

Food images were collected using the mobile food record (mFR^TM^) as part of the Food in Focus study, which was a community dwelling study of 45 adults (15 men and 30 women) between 21 and 65 years of age in a 7-day study period [35]. Pre-weighed food pack-outs were distributed to the participants and uneaten foods were returned and weighted. Briefly, participants captured images of each eating occasion over the entire period using the mFR^TM^. Providing known foods and amounts supported the objective of being able to identify the foods consumed and their amounts, which were used as ground truth for evaluating the proposed method. The food categories provided for breakfast, lunch, and dinner are listed in Table 1.

**Figure 1:**
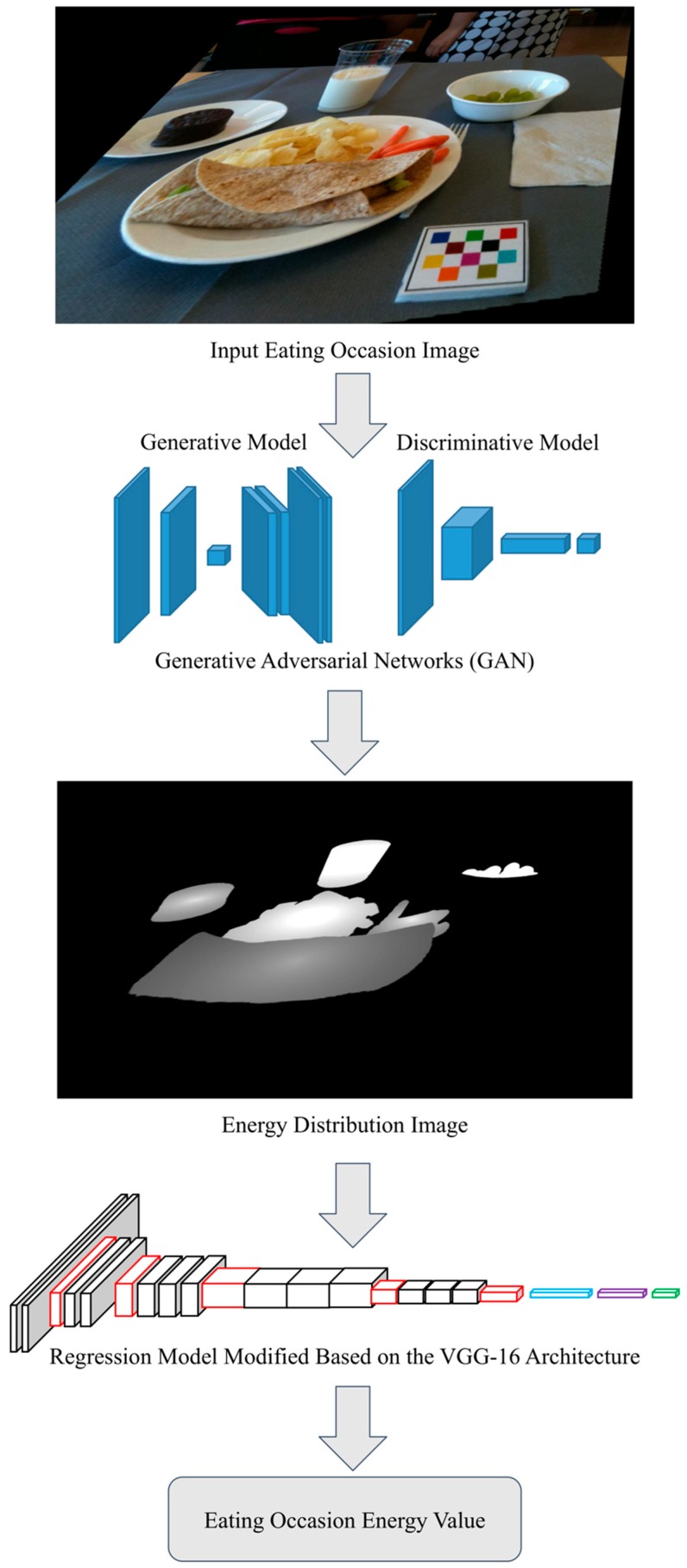
End-to-end system design of food energy estimation based on a single-view RGB eating occasion image.

Since there is no public data set available for training our generative model, the data set of image pairs, consisting of eating occasion images and corresponding energy distribution images, were constructed using the Food in Focus study. The purpose of this data set was to learn the mappings from food images to the food energy distribution images [33]. This data set was based on the ground truth food labels, segmentation masks, and energy information from the study where known foods and amounts were provided [35]. To build this data set, ground truth food labels, segmentation masks, food energy information, and the presence of the known size fiducial marker were required. To the best of our knowledge, we are the only group that has collected such a food image data set with all required information listed above. We used GAN [34] architecture to train the generative model for the task predicting the food energy distribution image, as GAN has shown impressive success in training generative models [27,28,29,36,37]. In addition, GAN is able to effectively reduce the adversarial space during training [34] compared to other generative models, such as Variational Autoencoders (VAEs) [38]. Our image-to-energy data set described in Section 2.1 could not cover all food types, eating scenes, and all possible food combinations. Therefore, GAN’s characteristic reducing adversarial space was important for our task, as it reduced the chance of the generative model overfitting on training image pairs. The energy value of the meal image is estimated based on the learned food energy distribution image by training a CNN. Figure 1 shows the design of the proposed end-to-end food energy estimation based on a single-view eating occasion image.

To train the GAN for the task of mapping eating occasion images to energy distribution images, eating occasion image and energy distribution image pairs were required. There is no device that can be used to directly capture the “energy distribution image”. We constructed the image-to-energy distribution data set using food images collected from the Food in Focus study [35]. Each food item and each eating occasion image were manually labeled and segmented in the data set. The ground truth energy information of each weighed food item in each eating occasion image was estimated using the energy values in the USDA Food and Nutrient Database for Dietary Studies.

**Figure 2:**
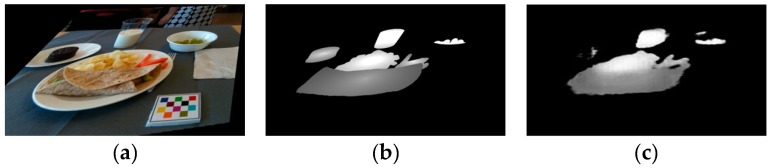
Learning image-to-energy translation using generative models. (**a**) Eating occasion image $\bar{I}$. (**b**) Ground truth energy distribution image $\bar{W}$. (**c**) Estimated energy distribution image $\tilde{W}$.

In order to construct the energy distribution image, we first detected the location of the fiducial marker [39]. A fiducial marker is a colored checkerboard, as shown in Figure 2a, which is included in each eating occasion scene image. The marker is used to correct the color of the acquired images to match the reference colors during food identification and for camera calibration in portion size estimation [40,41]. The image-to-energy distribution data set could not be constructed if any of the above components (ground truth food labels, segmentation masks, food energy information, and the presence of the known size fiducial marker) were missing.

With the reference of the known size fiducial marker, we removed the projective distortion in the original image using Direct Linear Transform (DLT) [42] based on the estimated homography matrix H to create a rectified image. Suppose I is the original eating occasion image; we denote $\hat{I}$ as the rectified image that is obtained: $\hat{I}=H^{-1}I$. Following the same rule of notation, for each food k and its associated segmentation mask $S_{k}$, the rectified segmentation can be expressed as: ${\hat{S}}_{k}=H^{-1}S_{k}$. For each pixel location $\left(\hat{i},\hat{j}\right)\in {\hat{S}}_{k}$, a scale factor ${\hat{w}}_{\hat{i},\hat{j}}$ is assigned to reflect the distance between the pixel location $\left(\hat{i},\hat{j}\right)$ to the centroid of the segmentation mask ${\hat{S}}_{k}$. Based on the scale factor ${\hat{w}}_{\hat{i},\hat{j}}$ assigned to each pixel location in ${\hat{S}}_{k}$, the weighted segmentation masks ${\hat{S}}_{k}$ can be projected back to the original pixel coordinates denoted as ${\bar{S}}_{k}$, where: ${\bar{S}}_{k}=H{\hat{S}}_{k}$, and learn the parameter $P_{k}$ such that:

(1) $$c_{k}=P_{k}\sum_{\forall \left(\bar{i},\bar{j}\right)\in {\bar{S}}_{k}}{\bar{w}}_{\bar{i},\bar{j}},$$

where $c_{k}$ is the ground truth energy associated with food k, $P_{k}$ is the energy mapping coefficient for ${\bar{S}}_{k}$, and ${\bar{w}}_{\bar{i},\bar{j}}$ is the energy weight factor at each pixel that makes up the ground truth energy distribution image. We can then update the energy weight factors ${\bar{w}}_{\bar{i},\bar{j}}$ in ${\bar{S}}_{k}$ as:

(2) $${\bar{w}}_{\bar{i},\bar{j}}=P_{k}\cdot {\bar{w}}_{\bar{i},\bar{j}},\forall \left(\bar{i},\bar{j}\right)\in {\bar{S}}_{k}.$$

Repeat the above process for all k∈{1,…,M}, where M is total number of food items in the eating occasion image, and then overlay all segments ${\bar{S}}_{k}$ onto the ground truth energy distribution image $\bar{W},$ whose size is the same as image $\bar{I}=H\hat{I}$. Here, we show a pair of image $\bar{I}$ and the energy distribution image $\bar{W}$, as shown in Figure 2a,b, accordingly. The estimated energy distribution image shown in Figure 2c is denoted as $\tilde{W}$, which is learned from training on pairs of images $\bar{I}$ and the ground truth energy distribution image $\bar{W}$.

### 2.2. Generative Adversarial Networks (GAN)

GAN architecture has shown impressive success in training generative models [27,28,29,36,37]. In GAN, two models are trained simultaneously: a generative model G that captures the data distribution, and a discriminative model D that determines the probability that a sample came from the training data rather than G [34]. The common analogy for the GAN architecture is a game between producing counterfeits (generative models) and detecting counterfeits (discriminative model) [34]. To formulate the GAN, we specified the cost functions. We use $\theta^{\left(G\right)}$ to denote the parameters of generative model G and $\theta^{\left(D\right)}$ to denote the parameters of discriminative model D. The generative model G attempts to minimize the cost function:

(3) $$J^{\left(G\right)}\left(\theta^{\left(D\right)},\theta^{\left(G\right)}\right)$$

where the discriminative model *D* attempts to minimize the cost function:

(4) $$J^{\left(D\right)}\left(\theta^{\left(D\right)},\theta^{\left(G\right)}\right)$$

In a zero-sum game, we have:

(5) $$J^{\left(G\right)}\left(\theta^{\left(D\right)},\theta^{\left(G\right)}\right)=-J^{\left(D\right)}\left(\theta^{\left(D\right)},\theta^{\left(G\right)}\right)$$

Therefore, the overall cost can be formulated as:

(6) $$J^{\left(D\right)}\left(\theta^{\left(D\right)},\theta^{\left(G\right)}\right)=-\frac{1}{2}E_{x\sim p_{data}}\left(x\right)\left[\log\;D\left(x\right)\right]-\frac{1}{2}E_{z\sim p_{z}\left(z\right)}\left[\log\;D\left(1-\left(G\left(z\right)\right)\right)\right]$$

where x is sampled from the true data $p_{data}$ and z is random noise generated by distribution $p_{z}$. The generative model takes z and generates fake sample G(z). The goal of the minimax game would then be:

(7) $$\underset{\theta \left(G\right)}{min}\;\underset{\theta \left(D\right)}{max}-J^{\left(D\right)}\left(\theta^{\left(D\right)},\theta^{\left(G\right)}\right)$$

Adversarial samples are those data which can easily lead neural networks to make mistakes. The GAN takes adversarial training samples by its nature, therefore, it could significantly reduce the adversarial space for the generative models to make mistakes. As a result, the use of GAN architecture can greatly reduce the training samples needed to model the statistical insights of the true data. During each update of the generative model G, the generated fake sample G(z) will become more like the true sample x. Therefore, after sufficient epochs of training, the discriminator D is unable to differentiate between the two distributions x and G(z) [34].

### 2.3. The Use of Conditional GAN (cGAN) for Image Mappings

We used conditional GAN (cGAN) [27] to estimate the energy distribution image [33], as cGAN is a natural fit for predicting an image output based on an input image. A cGAN attempts to learn the mapping from a random noise vector z to a target image y conditioned on the observed image x: G(x,z)→y. The objective of a cGAN can be expressed as:

(8) $${ℒ}_{cGAN}\left(G,D\right)=E_{x,y\sim p_{data}\left(x,y\right)}\left[\log\;D\left(x,y\right)\right]+E_{x\sim p_{data}\left(x\right),z\sim p_{z}\left(z\right)}\left[\log\;\left(1-D\left(x,G\left(x,z\right)\right)\right)\right]$$

Otherwise, an additional conditional loss ${ℒ}_{conditional}\left(G\right)$ [27] is added to further improve G(x,z)→y:

(9) $${ℒ}_{conditional}\left(G\right)=E_{x,y\sim p_{data}\left(x,y\right),z\sim p_{z}\left(z\right)\left[D\left(y,\;G\left(x,z\right)\right)\right],}$$

Common criteria used in D(y, G(x,z)) to measure the distance between y and G(x,z) are the $L_{2}$ distance [43]:

(10) $$D\left(y,\;G\left(x,z\right)\right)=\frac{1}{n}\overset{n}{\underset{i=1}{\sum }}{\left(y_{i}-G\left(x_{i},z_{i}\right)\right)}^{2}$$

the $L_{1}$ distance [27]:

(11) $$D\left(y,\;G\left(x,z\right)\right)=\frac{1}{n}\overset{n}{\underset{i=1}{\sum }}\left|\left(y_{i}-G\left(x_{i},z_{i}\right)\right)\right|$$

and a smooth version of the *L*1 distance:

(12) $$D\left(y,\;G\left(x,z\right)\right)=\{\begin{array}{c} \frac{{\left(y_{i}-G\left(x_{i},z_{i}\right)\right)}^{2}}{2}\;\;\;\;\;\;\;\;\;\;\;\;\;\;if\left|y_{i}-G\left(x_{i},z_{i}\right)\right|<1\\ \left|y_{i}-G\left(x_{i},z_{i}\right)\right|\;\;\;\;\;\;\;\;\;\;\;\;otherwise. \end{array}$$

So, the final objective [27,34] is:

(13) $$G^{*}=arg\;\underset{G}{min}\;\underset{D}{max}\;{ℒ}_{cGAN}\left(G,\;D\right)+\lambda {ℒ}_{conditional}\left(G\right)$$

where the generative model $G^{*}$ is used to estimate the energy distribution image $\tilde{W}$ based on the input eating occasion image $\bar{I}$.

### 2.4. Food Energy Estimation Based on Energy Distribution Images

**Figure 3:**
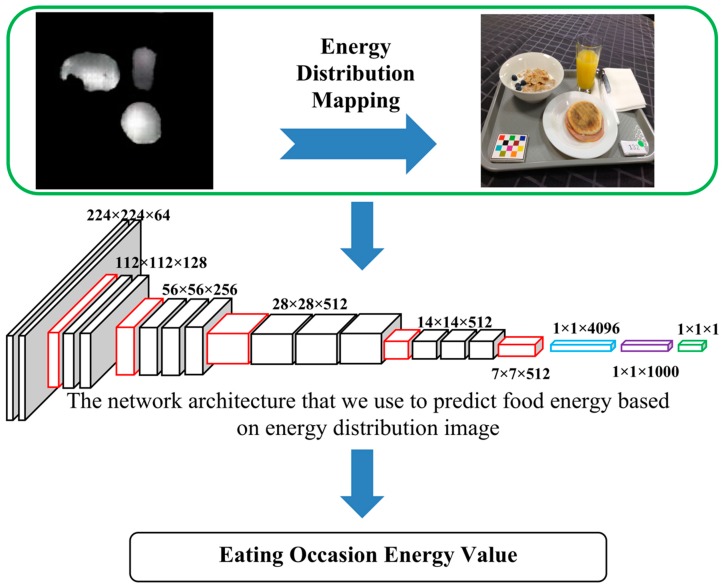
Estimating food energy of a meal based on predicted energy distribution image.

**Figure 4:**
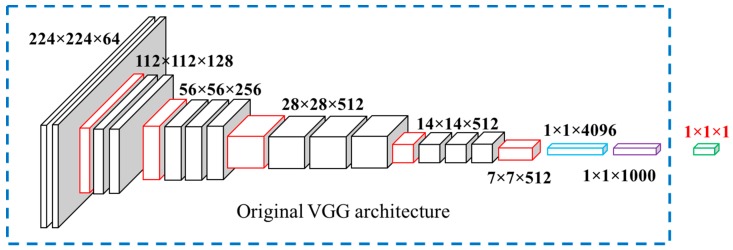
The network architecture used to predict food energy based on energy distribution image.

We were able to obtain the energy distribution image [33] for each RGB eating occasion image using generative model G trained by GAN. An example of an original food image and an estimated energy distribution image is shown in Figure 2a,c. Energy distribution images represent how food energy is distributed in the eating scene. Our goal was to estimate food energy (a numerical value) based on the estimated energy distribution image. This is essentially a regression task as shown in Figure 3. We used a CNN-based regression model to conduct the task of estimating energy from energy distribution images. For the regression model, we used a VGG-16-based [23] architecture, as shown in Figure 4. As VGG-16 has shown impressive results on object detection tasks, VGG-16 is sufficient for learning complex image features. We modified the original VGG-16 architecture and added an additional linear layer, as shown in Figure 4, so that the CNN-based architecture was suitable for the energy value regression task. Instead of using random initialization for VGG-16 and training from scratch, we used pre-trained weights of VGG-16 architecture on ImageNet [44]. The pre-trained weights are indicated in the dash bounding box in Figure 4. We used random initialization for the linear layer. We then fine-tuned the pre-trained weights of the VGG-16 network for energy value prediction task based on the building blocks of complex features originally learned from ImageNet [44]. With the regression model, we can predict the energy of the foods in a single-view eating occasion image.

## 3. Experimental Results

### 3.1. Learning Image-to-Energy Mappings

We used 202 food images [35] that were manually annotated with ground truth segmentation masks and labels which we used for training. Data augmentation techniques, such as rotating, cropping, and flipping, were used to expand the database. In total, there were 1875 paired images (an image pair contains one eating occasion image and its corresponding energy distribution image) used to train the cGAN and 220 paired images for testing.

Once the cGAN estimated the energy distribution image $\tilde{W}$, we could then determine the energy for a food image (portion size estimation) as:

(14) $$EstimatedEnergy=\underset{\forall \left(i,j\right)\in I}{\sum }\left({\tilde{W}}_{i,j}\right)$$

To compare the estimated energy image $\tilde{W}$ (Figure 2c) with the ground truth energy image $\bar{W}$ (Figure 2b), we defined the error between $\bar{W}$ and $\tilde{W}$ as:

(15) $$Energy\;Estimation\;Error\;Rate\;=\;\frac{\sum_{\forall \left(i,j\right)\in \bar{I}}\left({\tilde{W}}_{i,j}-{\bar{W}}_{i,j}\right)}{\sum_{\forall \left(i,j\right)\in \bar{I}}\left({\bar{W}}_{i,j}\right)}$$

**Figure 5:**
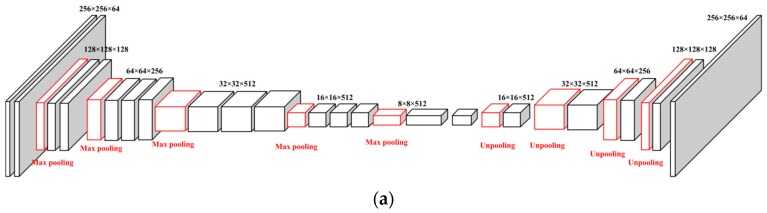

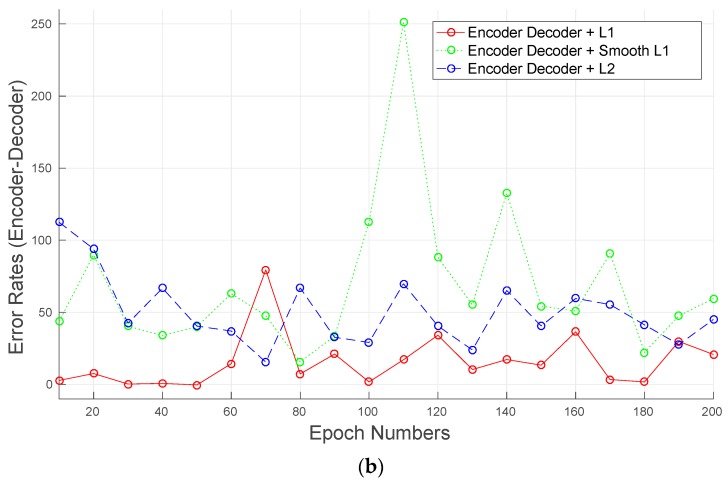
Generative model: encoder-decoder. (**a**) Architecture of encoder-decoder. (**b**) Error rate of encoder-decoder.

**Figure 6:**
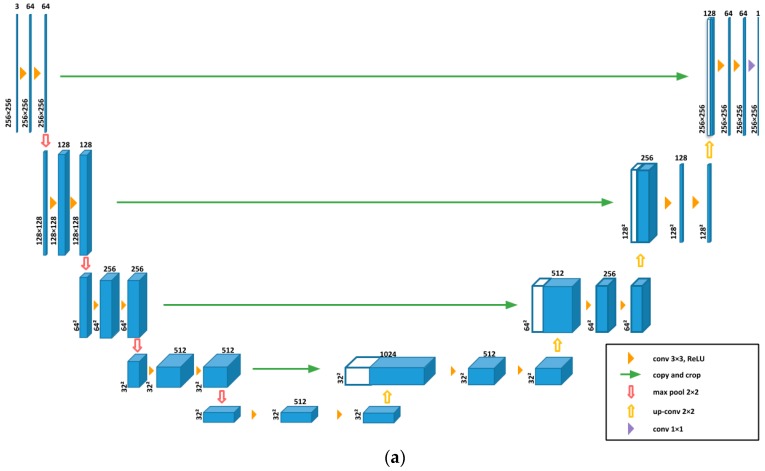

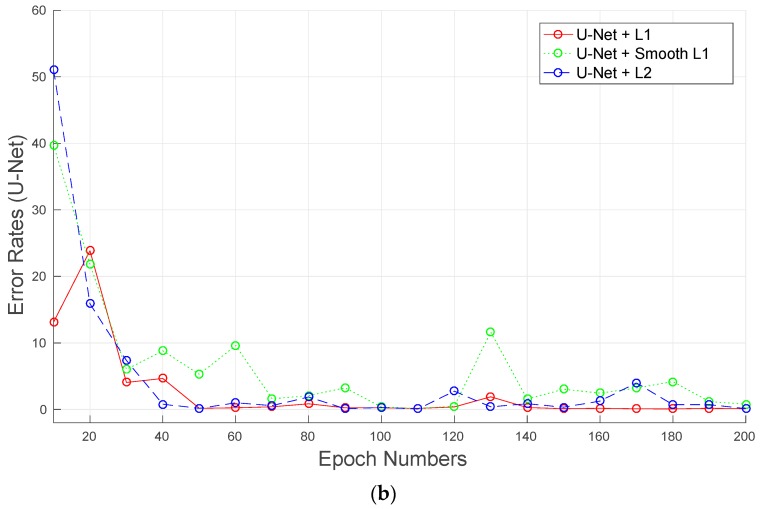
Generative model: U-Net. (**a**) Architecture of U-Net. (**b**) Error rate of U-Net.

We compared the energy estimation error rates at different epochs for the two different cGAN models we used, the encoder-decoder architecture (Figure 5) and the U-Net architecture (Figure 6). Compared to the encoder-decoder architecture (Figure 5), the U-Net architecture (Figure 6) was more accurate and stable. The reason is that information from the “encoder” can be directly copied to the “decoder” layers in the U-Net architecture to provide precise locations [45], which is an idea similar to ResNet [25].

We also compared the energy estimation error rates under different conditional loss settings, ${ℒ}_{conditional}\left(G\right)$, using U-Net. We used the batch size of 16 with λ=100 in Equation (13), the Adam [46] solver with initial learning rate α=0.0002, and momentum parameters $\beta_{1}=0.5$, $\beta_{2}=0.999$ [27]. We observed that distance measure D(y,G(x,z)) as defined in Equations (10)–(12) using the $L_{1}$ or $L_{2}$ norms is better than using smoothed $L_{1}$ norm. At epoch 200, the energy estimation error rates are 10.89% (using $L_{1}$ criterion) and 12.67% (using $L_{2}$ criterion), respectively. In the experiments, we included food types whose shapes are difficult to define (for example, fries). Predicting the energy for these food types is very challenging using a geometric-model-based approach [17].

### 3.2. Food Energy Estimation Based on Energy Distribution Images

**Figure 7:**
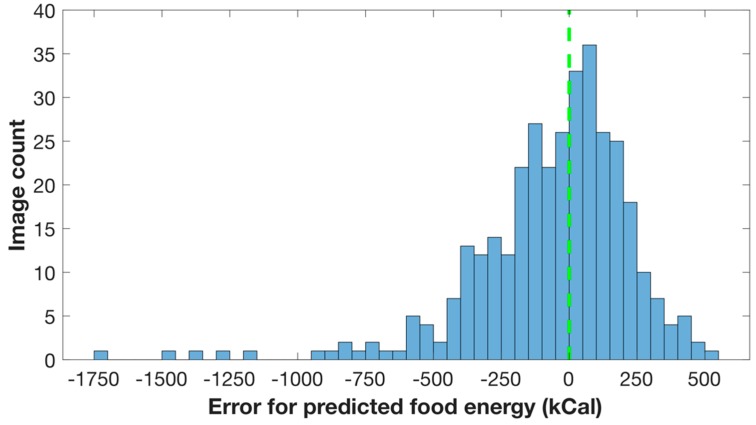
Error distribution of predicted food energy for all eating occasion images.

We predicted the food energy of each eating occasion image based on its energy distribution generated by generative model. The dimension for the predicted energy distribution image was 256 by 256. We resized the predicted energy distribution image from 256 by 256 to 224 by 224 to fit the input image size of VGG-16 architecture. To resize the output from generative model, we used OpenCV implementation of image resize, which is based on linear interpolation. The food energy estimation was then compared to the ground truth food energy from the Food in Focus study. We used 1390 eating occasion images also collected from the Food in Focus study [35], with ground truth food energy (kilocalories) for each food item in the eating occasion image. A total of 1043 of these eating occasion images were used for training and 347 of them for testing. The images selected for training and testing were selected by random sampling. All of the eating occasion images were captured by the users sitting naturally at a table. There were no extreme changes in the viewing angle. The errors for predicted food energy in Figure 7 are defined as:

(16) Error=Estimated Food Energy − Ground Truth Food Energy

**Figure 8:**
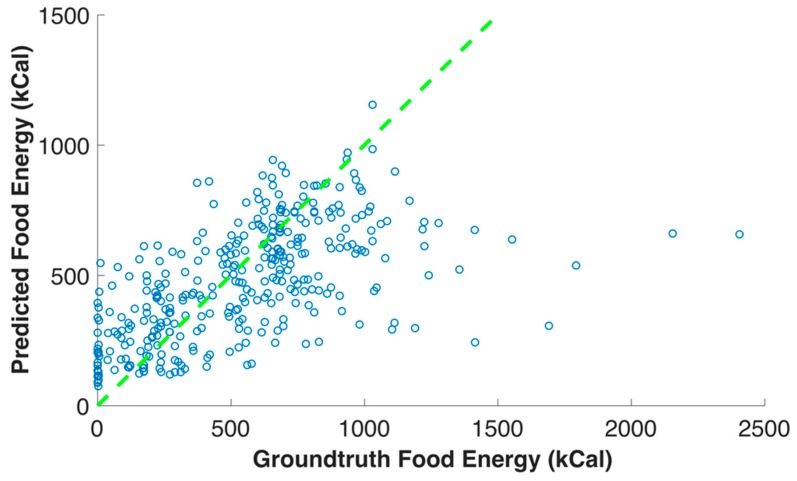
Relationship between the ground truth food energy and the food energy predicted for each eating occasion.

**Figure 9:**
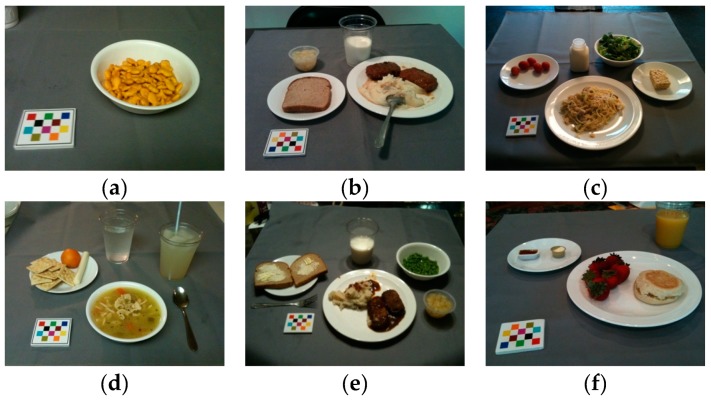
Examples of over-estimated food energy. (**a**) Ground truth energy: 287 kCal Predicted energy: 314 kCal Energy error: +27 kCal. (**b**) Ground truth energy: 520 kCal Predicted energy: 621 kCal Energy error: +101 kCal. (**c**) Ground truth energy: 653 kCal Predicted energy: 875 kCal Energy error: +222 kCal. (**d**) Ground truth energy: 498 kCal Predicted energy: 579 kCal Energy error: +81 kCal. (**e**) Ground truth energy: 705 kCal Predicted energy: 893 kCal Energy error: +188 kCal. (**f**) Ground truth energy: 354 kCal Predicted energy: 425 kCal Energy error: +71 kCal.

**Figure 10:**
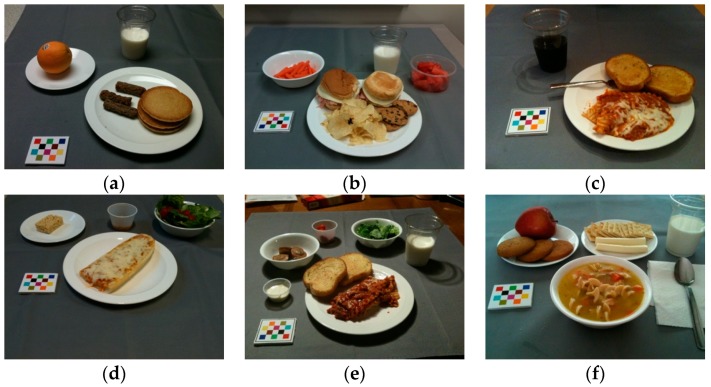
Examples of under-estimated food energy. (**a**) Ground truth energy: 542 kCal Predicted energy: 472 kCal Energy error: −70 kCal. (**b**) Ground truth energy: 990 kCal Predicted energy: 732 kCal Energy error: −258 kCal. (**c**) Ground truth energy: 508 kCal Predicted energy: 504 kCal Energy error: −4 kCal. (**d**) Ground truth energy: 508 kCal Predicted energy: 474 kCal Energy error: −34 kCal. (**e**) Ground truth energy: 749 kCal Predicted energy: 629 kCal Energy error: −120 kCal. (**f**) Ground truth energy: 1084 kCal Predicted energy: 708 kCal Energy error: −376 kCal.

Figure 8 shows the relationship between the ground truth food energy and the food energy estimation of the eating occasion images in the testing data set. The dash line in Figure 8 indicates the ground truth and estimated energy are the same, i.e., estimation error is equal to zero. Therefore, the points above this line are overestimated, and the points below this line are underestimated. Figure 9 and Figure 10 show examples of food energies the have been over- and underestimated, and we use “+” and “−” to indicate over- and underestimation, respectively. The average ground truth of an eating occasion image in the testing data set was 538 kilocalories. We observed that the estimation was more accurate for the eating occasion image with ground truth energy around average, when compared to those with extremely high or low ground truth energy, such as zero kilocalories. This is due to the fact that there were not sufficient eating occasion images in our data set with very high or low ground truth energy provided to the neural networks for training.

The error distribution of predicted food energies for 347 eating occasion images is shown in Figure 7. We found that the average energy estimation error was 209 kilocalories. An overestimation is displayed as a positive number. The average ground truth for all eating occasion images was 546 kilocalories, and the average ground truth for breakfast, lunch, and dinner eating occasion images was 531 kilocalories, 603 kilocalories, and 506 kilocalories, respectively. The average energy estimation error we obtained was 209 kilocalories, and the average energy estimation error for breakfast, lunch, and dinner eating occasion images was 204 kilocalories, 211 kilocalories, and 210 kilocalories, respectively. Several sample eating occasion images for overestimated food energy are shown in Figure 9, and eating occasion images for underestimated food energy are shown in Figure 10 accordingly.

## 4. Discussion

We have advanced the field of research for automatic food portion estimation by developing a novel food image based end-to-end system to estimate food energy using learned energy distribution images. The contributions of this work can be summarized as the following: We introduced a method for modeling the characteristics of energy distribution in an eating scene using generative models. Based on the predicted food energy distribution image, we designed a CNN-based regression model to estimate the energy value based on the learned energy distribution images. We designed and implemented a novel end-to-end system to estimate food energy based on a single-view RGB eating occasion image. The results were validated using data generated from the Food in Focus study using data from the 45 community-dwelling men and women between 21–65 years old consuming known foods and amounts over 7 days [35].

The advantage of our technique compared to a geometric model-based technique is that the system is training based. The pre-defined geometric models were limited to cover only certain types of food with known shapes, which is no longer an issue for training-based methods. In addition, the “energy distribution image” we introduced enabled us to first visualize how food energy estimation is spatially distributed across the eating scene (for example, regions of the image containing apple should have smaller weights due to lower energy (in kcal) compared to regions in the image containing cheese). Therefore, not only the final estimated numeric energy values could be used to analyze where the error may have come from, but also the intermediate results of the “energy distribution image” could be used.

As our end-to-end food portion estimation is a training based system, the limitation of the system is mainly determined by the training data. Expanding the training data set with a larger sample size, capturing images over a longer period of time, and more food types could improve the accuracy of automatic food portion estimation. For wider application, future studies need to include diverse eating styles and patterns, thus broadening the application of these methods to diverse population groups. These results point to the importance of controlled feeding studies using known foods and amounts. The results of such studies, on a wider scale, would contribute to wider application of these automated image-based methods with the benefit of improving accuracy of results. The use of an image-based system, such as TADA^TM^, which uses the mFR^TM^, is necessary for the automatic food portion estimation.

There are several reasons that may have led to the food energy estimation errors observed. Firstly, although we used 1875 paired food images to train the generative model using GAN architecture [33], the amount of food images did not cover all different eating occasions. Similarly, to train the regression model for numeric energy value prediction, 1043 eating occasion images were used where using more eating occasion images and food types could improve the accuracy of the end-to-end system. Secondly, when building the image-to-energy data set [33], the energy distribution images were synthetic images defined by handcrafted energy spread functions, rather than incorporating real 3D structures or depth information. Neither depth nor real 3D structure information was available when the study was conducted to capture eating occasion images [3]. To further improve the accuracy and address this challenge, we are currently investigating techniques to incorporate depth information into the end-to-end system where the 3D structures features of the foods in the images can also be learned by the neural networks.

## 5. Conclusions

In this work, we proposed a novel end-to-end system to directly estimate food energy using automatic food portion estimation from eating occasion images captured with an image-based system. Our system first estimated the image to energy mappings using a GAN structure. Based on the predicted food energy distribution image, we designed a CNN-based regression model to further estimate the energy value based the learned energy distribution images. To our knowledge, this method represents a paradigm shift in dietary assessment. The proposed method was validated using data collected by 45 men and women between 21–65 years old. We were able to obtain accurate food energy estimation with an average error of 209 kilocalories for eating occasion images collected from the Food in Focus study using the mFR^TM^. The training-based technique for end-to-end food energy estimation no longer requires fitting geometric models onto the food objects that may have issues scaling up, as we need a large amounts of geometric models to fit different food types in many food images. In the future, combining automatically detected food labels, segmentation masks, and contextual dietary information has the potential to further improve the accuracy of such end-to-end food portion estimation system.

## Figures and Tables

**Table 1:** Type of food items in eating occasion images separated by breakfast, lunch, and dinner.

Breakfast	Lunch	Dinner
Bagel	Apple	Apple
Banana	Bagel	Banana
English muffin	Carrot	Broccoli
Grape	Celery	Celery
Margarine	Cherry	Cherry
Mayonnaise	Chicken wrap	Doritos
Milk	Chocolate chip	Fruit cocktail
Orange	Ding Dong	Garlic bread
Orange juice	Doritos	Garlic toast
Pancake	Grape	Grape
Peanut butter	Ham sandwich	Lasagna
Ranch dressing	Mashed potato	Margarine
Saltines	Mayonnaise	Mashed potato
Sausage	Milk	Mayonnaise
Strawberry	Mustard	Milk
Syrup	No fat dressing	Muffin
Water	Noodle soup	Orange
Wheaties	Peas	Peas
Yogurt	Pizza	Ranch dressing
	Potato	Rice crispy bar
	Potato chip	Salad mix
	Ranch dressing	Strawberry
	Salad mix	String cheese
	Saltines	Tomato
	Snicker doodle	Water
	Strawberry	Watermelon
	String cheese	Wheat bread
	Tea	Yogurt
	Tomato	
	Water	
	Watermelon	
	Yogurt	
